# Impaired Cellular Bioenergetics Causes Mitochondrial Calcium Handling Defects in *MT-ND5* Mutant Cybrids

**DOI:** 10.1371/journal.pone.0154371

**Published:** 2016-04-25

**Authors:** Matthew McKenzie, Michael R. Duchen

**Affiliations:** 1 Centre for Genetic Diseases, Hudson Institute of Medical Research, Clayton, Melbourne, Victoria 3168, Australia; 2 The Department of Molecular and Translational Science, Monash University, Clayton, Melbourne, Victoria 3168, Australia; 3 Department of Physiology, University College London, Gower St, London, UK WC1E6BT; National Institute of Environmental Health Sciences, UNITED STATES

## Abstract

Mutations in mitochondrial DNA (mtDNA) can cause mitochondrial disease, a group of metabolic disorders that affect both children and adults. Interestingly, individual mtDNA mutations can cause very different clinical symptoms, however the factors that determine these phenotypes remain obscure. Defects in mitochondrial oxidative phosphorylation can disrupt cell signaling pathways, which may shape these disease phenotypes. In particular, mitochondria participate closely in cellular calcium signaling, with profound impact on cell function. Here, we examined the effects of a homoplasmic m.13565C>T mutation in *MT-ND5* on cellular calcium handling using transmitochondrial cybrids (ND5 mutant cybrids). We found that the oxidation of NADH and mitochondrial membrane potential (Δψ_m_) were significantly reduced in ND5 mutant cybrids. These metabolic defects were associated with a significant decrease in calcium uptake by ND5 mutant mitochondria in response to a calcium transient. Inhibition of glycolysis with 2-deoxy-D-glucose did not affect cytosolic calcium levels in control cybrids, but caused an increase in cytosolic calcium in ND5 mutant cybrids. This suggests that glycolytically-generated ATP is required not only to maintain Δψ_m_ in ND5 mutant mitochondria but is also critical for regulating cellular calcium homeostasis. We conclude that the m.13565C>T mutation in *MT-ND5* causes defects in both mitochondrial oxidative metabolism and mitochondrial calcium sequestration. This disruption of mitochondrial calcium handling, which leads to defects in cellular calcium homeostasis, may be an important contributor to mitochondrial disease pathogenesis.

## Introduction

Mitochondria provide the main source of energy in eukaryotic cells, oxidizing sugars, fats and amino acids to generate ATP by oxidative phosphorylation (OXPHOS). This series of enzymatic reactions is performed by five protein complexes (I-V) within the mitochondrial inner membrane. Complex I (NADH: ubiquinone oxidoreductase) and II (succinate-ubiquinone oxidoreductase) accept electrons from the TCA cycle, which are then passed to molecular oxygen via complexes III (ubiquinol: cytochrome *c* oxidoreductase) and IV (ferrocytochrome *c*: oxygen oxidoreductase). This transfer of electrons induces the pumping of protons out of the mitochondrial matrix by complexes I, III and IV to generate a mitochondrial membrane potential (Δψ_m_), which is subsequently used by complex V (F_o_F_1_-ATP synthetase) to generate ATP [1].

Mitochondria are unique organelles in that they contain their own circular genome. Mitochondrial DNA (mtDNA) encodes 13 polypeptides, all of which are protein subunits of the OXPHOS complexes I, III, IV and V. The mitochondrial genome also encodes the 12S and 16S rRNAs, as well as the 22 tRNAs that are specific for mitochondrial protein synthesis.

Mutations in mtDNA can disrupt OXPHOS function and cause mitochondrial disease, a diverse group of multi-systemic disorders that commonly affect the brain, heart and skeletal muscle. This includes syndromes such as mitochondrial encephalomyopathy, lactic acidosis and stroke-like episodes (MELAS), a heterogeneous disorder that presents with myopathy, encephalopathy and features of central nervous system involvement [2]. Conversely, some mtDNA mutations result in isolated symptoms, such as Leber Hereditary Optic Neuropathy (LHON), a form of acute blindness due to the specific loss of retinal ganglion cells in the optic nerve [3].

Apart from their essential role in generating ATP, mitochondria also perform many other important functions. In particular, mitochondria act as local calcium (Ca^2+^) buffers to tightly regulate intracellular Ca^2+^ concentration [4]. Mitochondrial utilize their Δψ_m_ to sequester Ca^2+^, allowing them to shape spatiotemporal cytosolic Ca^2+^ signaling within the cell [5]. The influx of Ca^2+^ into the mitochondria subsequently promotes the activity of three rate-limiting dehydrogenases of the citric acid cycle, which in turn upregulates OXPHOS [6]. In this way, mitochondrial calcium handling and OXPHOS function are tightly interlinked.

Apart from disrupting mitochondrial ATP production, mtDNA mutations have also been shown to cause mitochondrial Ca^2+^ handling defects. In fibroblasts from patients with MELAS, levels of ionized Ca^2+^ at rest are elevated compared to controls, with both Δψ_m_ and mitochondrial Ca^2+^ sequestration diminished [7]. Similarly, calcium homeostasis is altered in cybrids generated from the fibroblasts of patients with myoclonic epilepsy with ragged-red fibers (MERRF) [8].

We have previously shown that transmitochondrial cybrid cells carrying a homoplasmic m.13565C>T mtDNA mutation, which results in a p.Ser410Phe amino acid change in the complex I subunit ND5, have defects in mitochondrial respiration and a reduced Δψ_m_ [9]. Here, we examined the effects of this mutation on cell Ca^2+^ homeostasis and mitochondrial calcium handling, and found that mutant cybrid mitochondria have reduced levels of stored Ca^2+^ and a decreased capacity to accumulate increases in cytoplasmic Ca^2+^. These findings aid our understanding of the connection between OXPHOS dysfunction and mitochondrial Ca^2+^ homeostasis and how both can contribute to mitochondrial disease pathogenesis.

## Materials and Methods

### Cell lines and chemicals

All chemicals were from Sigma (St, Louis, MO, USA) unless otherwise specified. All cells were grown in RPMI 1640 Medium (ThermoFisher Scientific, Waltham, MA, USA) supplemented with 10% fetal bovine serum (ThermoFisher), 4.5 mg/mL glucose, 50 μg/mL uridine, and 1 mM pyruvate at 37°C and 5% CO_2_ unless otherwise specified.

Human mitochondrial cybrids used in this study were generated as previously described using mtDNA-less 143B osteosarcoma cells (ρ^0^) as the nuclear donors [9]. Cybrids contained either control wild-type mtDNA (CON) or a homoplasmic m.13565C>T mutation in the *MT*-*ND5* gene (ND5) which was captured from fibroblasts from a patient with mitochondrial encephalomyopathy and lactic acidosis with stroke-like episodes (MELAS) [9].

### Fluorescent Imaging of Cell Calcium

For measurements in the presence of calcium, cells were incubated in buffer containing 156 mM NaCl, 3 mM KCl, 2 mM MgSO_4_, 1.25 mM KH_2_PO_4_, 10 mM D-glucose, 2 mM CaCl_2_, 10 mM HEPES pH 7.35 (Record Solution, RS), 5 μg/mL fura-2 AM (ThermoFisher) and 0.005% pluronic (ThermoFisher) for 30 min in RS. Cells were then washed with 1xPBS before imaging in RS, with 10 μM ionomycin or 5 mM 2-deoxy-D-glucose (2DG) added as indicated.

For measurements in the absence of calcium, cells were incubated in buffer containing 156 mM NaCl, 3 mM KCl, 2 mM MgSO_4_, 1.25 mM KH_2_PO_4_, 10 mM D-glucose, 10 mM HEPES pH 7.35, 0.1 mM EGTA (Ca^2+^ free RS), 5 μg/mL fura-2 AM and 0.005% pluronic for 30 min. Cells were then washed with 1xPBS before imaging in Ca^2+^ free RS, with additions of 1 μM thapsigargin and 10 μM ionomycin added as indicated.

Fluorescent images were captured on a Nikon epifluorescence inverted microscope with a 40X objective. A xenon arc lamp with 10 nm band-pass filters centered at 340 and 380 nm was used for excitation (Cairn Research, Kent, UK), with emitted light passing through a 515 nm long-pass filter to an interline transfer cooled CCD camera (Orca ER, Hamamatsu). Images were digitized to 12-bit and analyzed using Kinetic Imaging software (Liverpool, UK).

### Fluorescent Imaging of Mitochondrial Calcium and Mitochondrial Membrane Potential

Mitochondrial membrane potential (Δψ_m_) was measured by incubating cells in RS with 20 nM tetramethylrhodamine, methyl ester, perchlorate (TMRM) (ThermoFisher) and 10 μM verapamil (which is required to inhibit TMRM export from the cell on the multidrug transporter). Cells were allowed to equilibrate the dye for at least 45 min at room temperature before imaging. Additions of 2 mM cyanide (CN^-^) or 10 μM carbonyl cyanide p-trifluoromethoxyphenylhydrazone (FCCP) were made as indicated.

For simultaneous measurement of Δψ_m_ and mitochondrial calcium [Ca^2+^]_m_, cells were incubated with 5 μg/mL fluo-4 AM (ThermoFisher), 0.005% pluronic, 20 nM TMRM and 10 μM verapamil for 45 min in RS. Cells were washed in Ca^2+^ free Hank’s buffered salt solution (HBSS, with 500 μM EGTA), permeabilized with 25 μg/mL digitonin in 6 mM NaCl, 130 mM KCl, 7.8 mM MgCl_2_, 1 mM KH_2_PO_4_, 0.4 mM CaCl_2_, 2 mM EGTA, 10 mM HEDTA, 2 mM malate, 2 mM glutamate, 2 mM ADP, 20 mM HEPES pH 7.35 (intracellular medium, IM), then left to equilibrate in 200 nM TMRM and 1 μM thapsigargin in IM. Aliquots of 0.4 mM CaCl_2_ were added to the cells at 3.5 min intervals. Final free Ca^2+^ ion concentration [Ca^2+^] was calculated using Chelator software [10].

Δψ_m_ and fluo-4 calcium measurements were made by acquiring images with a Carl Zeiss 510 inverted laser scanning confocal microscope (Oberkochen, Germany). TMRM and fluo-4 were excited using 543 nm He-Ne and 488 nm argon laser lines, respectively. NADH autofluorescence was measured using the 362 nm UV laser line. Images were analyzed using Carl Zeiss or Kinetic Imaging software, with mitochondrial calcium measured by selecting regions of interest that specifically co-localized with mitochondrial TMRM signals.

Statistical analyses for all experiments were performed using imaging data from three separate experiments, with significant differences determined using Student's two-tailed *t*-tests.

## Results

### NADH oxidation by Complex I is reduced in ND5 mutant cybrids

**Fig 1:**
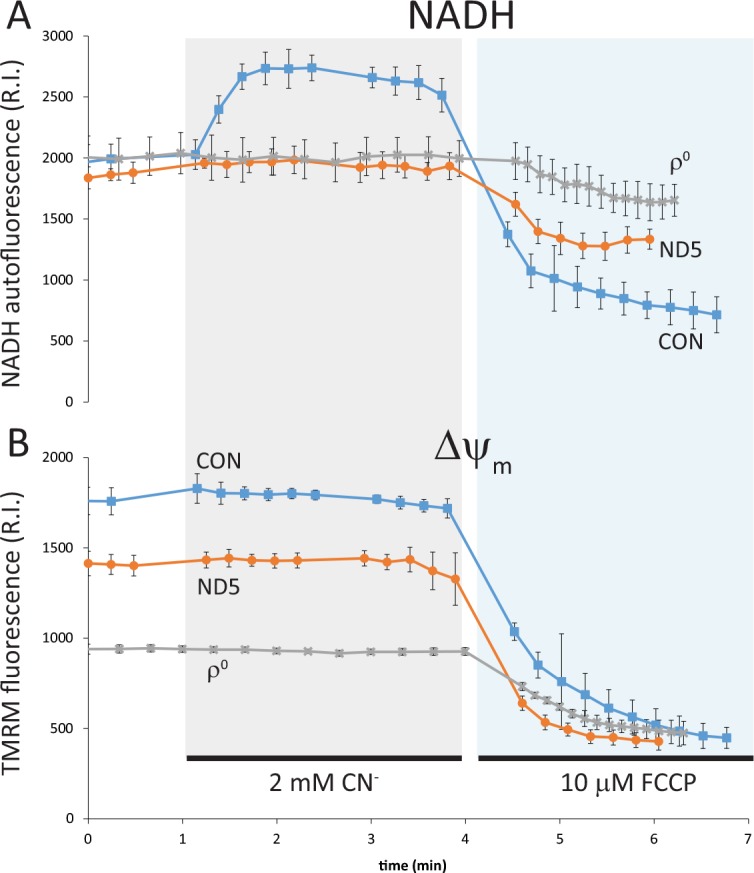
NADH oxidation and Δψ_m_ are reduced in ND5 mutant cybrids. NADH oxidation (NADH autofluorescence) and Δψ_m_ (TMRM fluorescence) were measured concurrently by confocal microscopy. (A) In control (CON) cybrids, inhibition of respiration with cyanide (CN^-^) increased NADH autofluorescence, reflecting a decrease in NADH oxidation. Conversely, CN^-^ had no effect on NADH oxidation in ND5 mutant cybrids or ρ^0^ cells. Stimulation of respiration with the uncoupler FCCP significantly increased NADH oxidation in CON cybrids, induced a small increase in NADH oxidation in ND5 mutant cybrids, but did not change NADH oxidation in ρ^0^ cells. (B) CN^-^ did not cause any significant change in Δψ_m_ in CON cybrids, ND5 mutant cybrids or ρ^0^ cells, whereas FCCP collapsed Δψ_m_ in all cell types. Data is mean ± s.d. n = 3.

We first examined the impact of the m.13565C>T mutation on mitochondrial function. Measurements of NADH ‘auto’ fluorescence reflects the redox balance of the NAD+/NADH pool, as only NADH is fluorescent. We explored the relationship between mitochondrial NADH redox state and Δψ_m_ by inhibiting cytochrome *c* oxidase (Complex IV) with cyanide (CN^-^), which blocks electron flow through the respiratory chain. In the control cybrid (CON), CN^-^ treatment caused the inhibition of NADH oxidation by Complex I, as observed by an increase in NADH autofluorescence (Fig 1A). However, blocking electron flow with CN^-^ did not collapse Δψ_m_, as the F_o_F_1_-ATP synthetase switches to function in reverse, maintaining Δψ_m_ [11] (Fig 1B).

Blockade of Complex IV with CN^-^ in the ND5 mutant cybrid did not alter NADH oxidation, suggesting that at rest the rate of NADH oxidation by complex I is very low (Fig 1A). Δψ_m_ in ND5 mutant cybrids, which is lower than in control cybrids, was also unchanged upon the addition of CN^-^, as it is maintained by reverse function of the F_o_F_1_-ATPase as previously shown [9] (Fig 1B).

As ρ^0^ cells do not have functional OXPHOS complexes to generate a Δψ_m_, they maintain a potential through the reversal of both the ANT and an incomplete F_o_F_1_-ATPase [9, 12]. Thus, the addition of CN^-^ had no effect either on NADH oxidation or Δψ_m_ in ρ^0^ cells (Fig 1A and 1B).

The protonophore carbonyl cyanide p-trifluoromethoxyphenylhydrazone (FCCP) will always collapse any potential based on proton distribution. In the control cybrid (CON), FCCP caused a collapse of Δψ_m_ and the rapid oxidation of NADH (indicated by a decrease in NADH autofluorescence) as respiration is stimulated (Fig 1B). NADH oxidation increased modestly in ND5 mutant cybrids after depolarization with FCCP, suggesting that there is a small reserve respiratory capacity when stimulated (Fig 1A). This result concurs with our previous findings, where the ratio of uncoupled/coupled respiration (as an indicatory of reserve respiratory capacity) was only 1.25 in ND5 mutant cybrid cells (compared to a ratio of 2.0 in control cybrid cells (p<0.05)) [9].

FCCP did not stimulate NADH oxidation in ρ^0^ cells (Fig 1A), despite collapsing the small Δψ_m_ (Fig 1B), confirming the absence of functional OXPHOS in these cells.

### Levels of stored calcium are reduced in ND5 mutant cybrids

**Fig 2:**
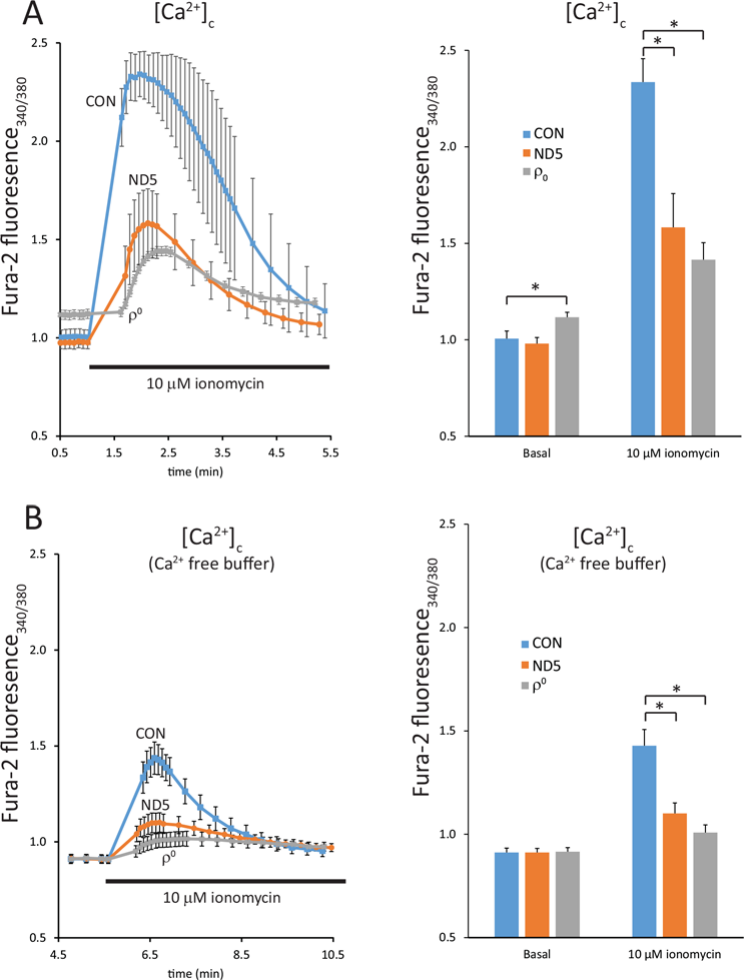
ND5 mutant cybrids exhibit reduced levels of stored calcium. Free cytoplasmic calcium [Ca^2+^]_c_ was measured using the ratiometric dye fura-2 AM on an epifluorescence imaging system. Increases in [Ca^2+^]_c_ were significantly lower in ND5 mutant cybrids and ρ^0^ cells than in control (CON) cybrids after the addition of the calcium ionophore ionomycin in either calcium-containing (A) or a calcium-free buffer (B). Data is mean ± s.d. n = 3. *p<0.05.

We used the ratiometric dye fura-2 to perform a quantitative comparison of cytosolic free Ca^2+^ [Ca^2+^]_c_ in control (CON) and ND5 mutant cybrids in real time. At rest in physiological saline (RS, with 2 mM Ca^2+^), the [Ca^2+^]_c_ in control (CON) and ND5 mutant cybrids was the same, whereas resting [Ca^2+^]_c_ was significantly higher in ρ^0^ cells (p<0.05) (Fig 2A). This is most likely a result of impaired Ca^2+^ clearance mechanisms due to the ATP depletion observed in this cell type [12, 13]. The Ca^2+^ ionophore ionomycin, at a concentration of 10 μM, releases Ca^2+^ preferentially from the endoplasmic reticulum (ER) and the mitochondria into the cytosol. Ionomycin treatment revealed significantly less stored Ca^2+^ in ND5 mutant cybrids (45.3 ± 11.0% of control (CON) cybrids, p<0.05) (Fig 2A). Stored Ca^2+^ in ρ^0^ cells was also significantly less than in control cybrids (22.4 ± 6.2%, p<0.05) (Fig 2A).

We also examined the release of stored Ca^2+^ in the absence of extracellular Ca^2+^ (Ca^2+^ free RS) (Fig 2B). Under these conditions, there was no significant difference in the levels of resting [Ca^2+^]_c_ in control (CON) cybrids, ND5 mutant cybrids or ρ^0^ cells (Fig 2B). However, the addition of ionomycin resulted in a similar pattern of Ca^2+^ release, in that the increase in [Ca^2+^]_c_ was significantly reduced in ND5 cybrids (36.6 ± 4.6%, p<0.05) and ρ^0^ cells (17.9 ± 3.7%, p<0.05) compared to the controls (Fig 2B). The total increase in [Ca^2+^]_c_ induced by ionomycin was reduced in each cell type compared to our findings presented in Fig 2A, as would be expected in the absence of free extracellular Ca^2+^ (Fig 2B).

We next used thapsigargin to measure the release of Ca^2+^ specifically from the ER, followed by ionomycin, which will then release calcium sequestered in the mitochondria. Thapsigargin inhibits the Ca^2+^-ATPase, causing depletion of ER Ca^2+^ and an increase in [Ca^2+^]_c_. Subsequent addition of ionomycin, in the absence of extracellular free Ca^2+^, will then release Ca^2+^ from any remaining intracellular pool, which will be dominated by the mitochondria.

**Fig 3:**
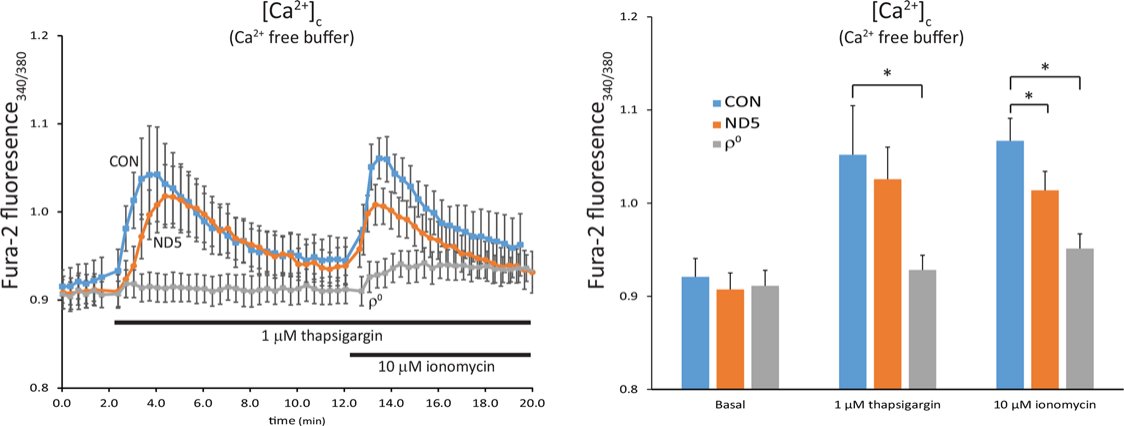
ND5 mutant mitochondria have reduced levels of stored calcium. Free cytoplasmic calcium [Ca^2+^]_c_ was measured using the ratiometric dye fura-2 AM on an epifluorescence imaging system. Thapsigargin was used to block the Ca^2+^-ATPase to release endoplasmic reticulum (ER) calcium stores, followed by the addition of ionomycin to release mitochondrial calcium. Calcium release from ER stores was not different between control (CON) cybrids and ND5 mutant cybrids, but was significantly reduced in ρ^0^ cells. However, calcium release from mitochondrial stores was significantly lower in ND5 mutant cybrids lower (66.4 ± 15.8%) that CON cybrids. Stored mitochondrial calcium was also significantly lower in ρ^0^ cells compared to CON cybrids (21.3 ± 11.7%). Data is mean ± s.d. n = 3.*p<0.05.

Cells were incubated in Ca^2+^ free buffer, followed by the addition of thapsigargin. This resulted in the release of Ca^2+^ from ER stores in both control (CON) cybrids and ND5 mutant cybrids (Fig 3). Although this Ca^2+^ release was both reduced and delayed in ND5 mutant cybrids, neither was statistically significant. In comparison, ρ^0^ cells showed no significant release of ER Ca^2+^, suggesting that the ATP depletion observed in these cells disrupts ER Ca^2+^ uptake [12, 13] (Fig 3).

Subsequent addition of ionomycin released Ca^2+^ from the mitochondria in both control (CON) and ND5 mutant cybrids, however, stored Ca^2+^ in ND5 mutant mitochondria was significantly lower (66.4 ± 15.8%, p<0.05) (Fig 3). Releasable Ca^2+^ in ρ^0^ cell mitochondria was also significantly lower than the control cybrid (21.3 ± 11.7%, p<0.05) (Fig 3).

### Mitochondrial calcium buffering power is significantly reduced in ND5 mutant cybrids

**Fig 4:**
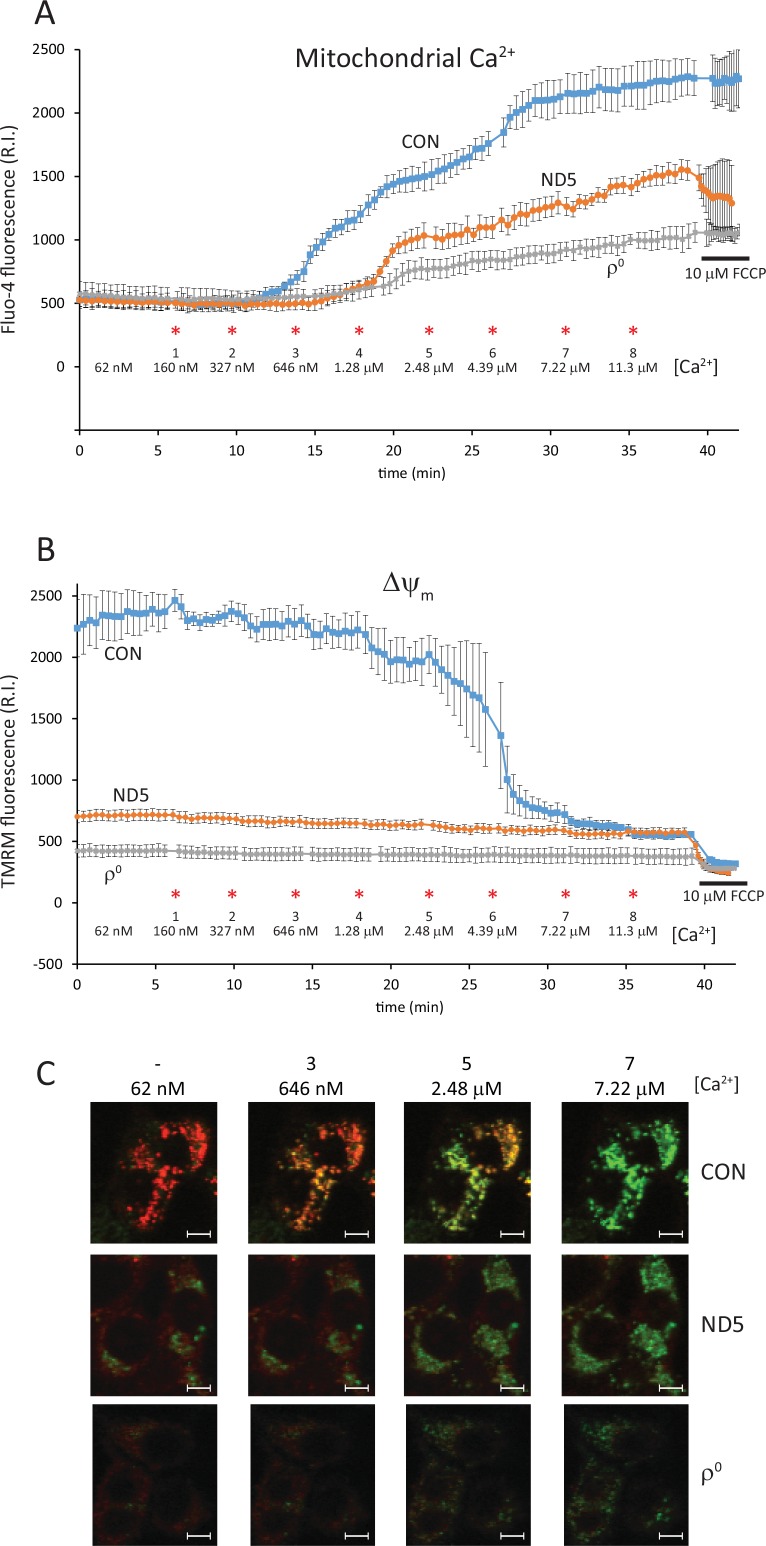
ND5 mutant mitochondria have a reduced ability to buffer calcium. Mitochondrial calcium [Ca^2+^]_m_ (A) and Δψ_m_ (B) were measured concurrently by confocal microscopy using fluo-4 and TMRM in digitonin permeabilized cells. Exogenous calcium was added in sequential aliquots at the concentrations and times indicated (*). Control (CON) cybrid mitochondria were able to buffer more calcium than both ND5 mutant cybrid mitochondria and ρ^0^ cell mitochondria. Increases in [Ca^2+^] up to 2.48 μM were buffered by CON cybrid mitochondria, with a step-wise decrease (followed by recovery) of Δψ_m_ evident. Δψ_m_ was lower in both ND5 mutant cybrids and ρ^0^ cells, and was not affected by increases in [Ca^2+^]. (C) Confocal images of CON cybrids, ND5 mutant cybrids and ρ^0^ cells showing the simultaneous staining of [Ca^2+^]_m_ (fluo-4, green) and Δψ_m_ (TMRM, red). The number of the calcium aliquot and the [Ca^2+^] are indicated. Data is mean ± s.d. n = 3.White scale bars = 10 μm.

In order to measure the ability of the mitochondria to buffer increases in Ca^2+^, cybrids were loaded with fluo-4 and then permeabilized with digitonin in a cytosol-like buffer (intracellular medium, IM) containing 62 nM free Ca^2+^. In addition, thapsigargin was added to deplete ER Ca^2+^ stores. Under these conditions, fluo-4 is lost from both the cytosol and ER, with the only dye remaining trapped in the mitochondria (Fig 4C). Aliquots of Ca^2+^ were then added directly to the cells and mitochondrial calcium [Ca^2+^]_m_ and Δψ_m_ (using TMRM) were measured simultaneously by confocal microscopy.

Increasing [Ca^2+^] to 327 nM caused no change in [Ca^2+^]_m_ in control cybrids (Fig 4A), although slight depolarization and recovery of Δψ_m_ is evident (Fig 4B). Further increases to [Ca^2+^] in control cybrids increased [Ca^2+^]_m_ progressively, producing a large depolarization of Δψ_m_ at 2.48 μM [Ca^2+^]_c_ (Fig 4B). At higher [Ca^2+^], an intact Δψ_m_ was still evident, as the addition of 10 μM FCCP still induced the collapse of Δψ_m_ (Fig 4B).

In contrast, mitochondrial calcium accumulation was reduced significantly in ND5 mutant cybrids, and no increase in [Ca^2+^]_m_ was measurable until [Ca^2+^] reached 1.28 μM (Fig 4A). At a [Ca^2+^] of 2.48 μM, the increase in [Ca^2+^]_m_ was only 47.5% of that seen in control cybrid mitochondria (Fig 4A). This reduced Ca^2+^ uptake by ND5 mutant mitochondria was also observed at the highest [Ca^2+^] tested, 11.3 μM, with [Ca^2+^]_m_ only 59.1% of control cybrid mitochondria (Fig 4A).

We have previously observed that Δψ_m_ in resting, intact ND5 mutant cybrids was significantly lower than in matching control cybrids (72%, p<0.05) [9]. In permeabilized cells, the TMRM intensity in ND5 mutant mitochondria was only 31.3% (p<0.05) of control cybrid mitochondria at a [Ca^2+^] of 62 nM (Fig 3B). This further decrease in ND5 mutant Δψ_m_ is likely a result of the diminished ATP pool after permeabilization, as glycolytically derived ATP is required (in part) to maintain Δψ_m_ in ND5 mutant mitochondria [9].

ρ^0^ mitochondria exhibited a very limited capacity for Ca^2+^ accumulation, with [Ca^2+^]_m_ only 24% of control values when [Ca^2+^] was elevated to 2.48μM (Fig 4A). Δψ_m_ did not change at all in ρ^0^ cells with increases in [Ca^2+^] (Fig 4B), however the addition of FCCP still elicited a small depolarization (Fig 4B). Of note, Δψ_m_ in ND5 mutant mitochondria was higher than in ρ^0^ mitochondria after permeabilization, suggesting that the reduced respiratory activity in ND5 mutant mitochondria can maintain a modest Δψ_m_ in the absence of glycolytically derived ATP (Fig 4B).

Examples of images from which the simultaneous measurements of [Ca^2+^]_m_ (with fluo-4; green) and Δψ_m_ (with TMRM; red) were made at various [Ca^2+^] in control (CON) cybrids, ND5 mutant cybrids and ρ^0^ cells are shown (Fig 4C).

### Calcium buffering in ND5 mutant cybrids is dependent on ATP derived from glycolysis

We have previously shown that ND5 mutant cybrids primarily use glycolysis to generate ATP, and that this ATP is utilized to partially maintain Δψ_m_ [9]. Here, we examined the effects of inhibiting glycolysis using 5mM 2-deoxy-D-glucose (2DG) on [Ca^2+^]_c_.

**Fig 5:**
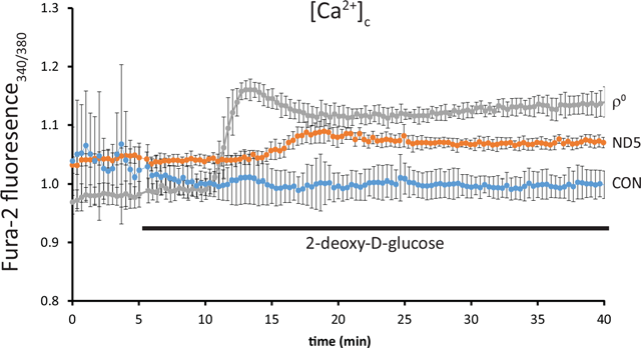
Calcium buffering in ND5 mutant cybrids is dependent on glycolytically-derived ATP. Free cytoplasmic calcium [Ca^2+^]_c_ was measured using the ratiometric dye fura-2 AM on an epifluorescence imaging system. Inhibition of glycolysis with 2-deoxy-D-glucose (2DG) did not affect [Ca^2+^]_c_ in control (CON) cybrids. Conversely, a significant increase in [Ca^2+^]_c_ was observed in ρ^0^ cells after approximately 5 min 2-deoxy-D-glucose treatment. [Ca^2+^]_c_ also increased significantly in ND5 mutant cybrids after approximately 12 min treatment, however this increase in [Ca^2+^]_c_ was smaller than in ρ^0^ cells. Data is mean ± s.d. n = 3.

In control (CON) cybrids, [Ca^2+^]_c_ remained unchanged up to 35 min after 2DG administration (Fig 5). However, in ND5 mutant cybrids, [Ca^2+^]_c_ began to increase after approximately 10 min of 2DG treatment and remained significantly higher in ND5 than in control (CON) cybrids up to 35 min of treatment (Fig 5, p<0.05). In contrast, [Ca^2+^]_c_ increased significantly in ρ^0^ cells after only 5 min 2DG treatment (p<0.05), followed by a slight reduction for the remainder of the experiment (Fig 5).

## Discussion

The relationship between mitochondrial metabolism and calcium signaling was first recognized in the early 1960’s, when it was discovered that isolated mitochondria could take up calcium [14]. Since that time, it has become apparent that mitochondrial calcium handling is important in a range of different cellular functions, including neuronal signaling and induction of cell death [15]. Furthermore, it is now clear that defects in mitochondrial metabolism can disrupt cellular calcium homeostasis and contribute to human disease pathology. Various pathogenic mtDNA point mutations associated with mitochondrial disease have been shown to affect mitochondrial calcium handling. In fibroblasts from patients with MELAS, Δψ_m_ was found to be reduced, with an associated increase in basal cytosolic Ca^2+^ [7]. Similarly, cybrid cells carrying the m.3243A>G mutation associated with MELAS were also found to exhibit increased cytosolic calcium levels [16]. Mitochondrial calcium homeostasis is also altered in cybrids carrying the m.8344A>G tRNA^Lys^ mutation associated with myoclonic epilepsy with ragged-red fibers (MERRF) and have been shown to have a decreased capacity to take up cytosolic calcium [8, 17].

In ND5 mutant cybrid mitochondria, we observed a decrease in both the mitochondrial calcium content and their ability take up increases in exogenous calcium. The homoplasmic m.13565C>T mtDNA mutation in these cybrids causes a respiratory defect that results in a reduced Δψ_m_, which is maintained in-part by glycolytically-derived ATP which fuels the reverse action of the ATP synthase [9]. Blocking glycolysis reduces Δψ_m_ in ND5 mutant mitochondria further, with a subsequent elevation of cytosolic calcium levels. These findings support the notion that efficient mitochondrial metabolism is not only important for generating ATP, but that it is also critical for regulating cellular calcium homeostasis. Indeed, the calcium handling defect in ND5 mutant cybrid mitochondria appears to be directly related to their reduced Δψ_m_ and their reliance on glycolytically derived ATP. However, their inability to accumulate calcium may also be due to defective calcium influx mechanisms. The inherent respiratory defect in these cells will result in a diminished electrogenic force across the mitochondrial inner membrane, which in turn may reduce calcium influx via the mitochondrial calcium uniporter [18].

When [Ca^2+^] reached 2.48 μM in control cybrids, a large depolarization of Δψ_m_ was observed, indicating the initiation of mitochondrial membrane permeability transition. Interestingly, a large membrane depolarization was not observed in either the ND5 mutant cybrids or ρ^0^ cells. This may be due to the fact that resting Δψ_m_ in both these cell types is lower than Δψ_m_ in control cybrids following membrane permeability transition. Nevertheless, it is possible that mitochondrial permeability transition, or low conductance pore opening, is occurring in both ND5 mutant cybrids and ρ^0^ cells at high [Ca^2+^].

Calcium is particularly important in neuronal cells, where calcium signaling is critical for signal processing. In these cells, defects in mitochondrial metabolism will have severe functional consequences, as the reduction in ATP generation will lead to elevated cytoplasmic calcium levels that disturb cell signaling pathways, confound normal signal processing, and may even promote cell death via calcium overload and/or excitotoxicity [19]. In neurons differentiated from mouse embryonic stem cells, which carry mtDNA mutations that disrupt OXPHOS complex I or IV activity, stimulation with glutamate resulted in calcium transients that were no different to control cybrid neurons [20]. However, repeated stimulation in the mutant neurons resulted in calcium transients that decayed increasingly slowly, resulting in elevated cytoplasmic calcium levels [20]. This loss of calcium regulation has the potential to disrupt neuronal transmitter release, long-term potentiation and depression, as well as developmental remodeling [20].

It has long been postulated that disruption of cell death signaling pathways may be a contributing factor to mtDNA disease pathogenesis [21]. Furthermore, calcium homeostasis may play an important role in this process. Cybrid cells carrying the m.8344A>G mtDNA mutation associated with MERFF were shown to be hypersensitive to staurosporine-induced cell death, and that this hypersensitivity was mediated by the calcium-dependent activation of calpains [22]. Cybrids carrying the m.8993T>G NARP mutation also display increased sensitivity to cell death induction, in this case by thapsigargin [17]. However, MERRF cybrids are protected from thapsigargin-induced cell death, even though both the NARP and MERFF mutant cybrids display similar defects in mitochondrial calcium uptake [17]. This suggests that other factors are involved in regulating the response of these two mutant cybrids to ER stress. Indeed, NARP mutant cybrids showed a significant increase in free radical generation [17] and also disturbed actin cytoskeleton organization, which subsequently disrupts capacitative calcium entry [23].

The ND5 mutant cybrids showed a reduced ability to generate ATP and exhibit defects in mitochondrial calcium accumulation, two features which serve to compound mitochondrial disease pathology. Of note, the m.13565C>T *MT*-*ND5* mutation studied here was originally isolated from a MELAS patient who also carries a mutation in *POLG*, which encodes the catalytic subunit of the mitochondrial DNA polymerase [24]. Nevertheless, our transmitochondrial cybrid studies clearly show that the *MT*-*ND5* mutation disrupts both mitochondrial respiratory function and calcium handling, and suggests that the *MT*-*ND5* mutation may play a role in modulating the disease phenotype in conjunction with the *POLG* mutation.

Changes in cytosolic calcium concentration signal the mitochondria to match energy supply with demand, in particular by regulating mitochondrial dehydrogenase activity [25–28]. However, failure of mitochondria to accumulate calcium, as in ND5 mutant cybrids, will result in the loss of TCA cycle enzyme stimulation, a lower than required respiratory rate and subsequently a reduced Δψ_m_. This will have a profound impact on cell metabolism, with energetic failure or collapse of Δψ_m_ leading to cell death [6]. As such, mitochondrial calcium handling is an important factor to consider when investigating mitochondrial disease pathogenesis. Furthermore, the future design of therapies for treating mitochondrial disease will need to address not only the energetic deficits caused by OXPHOS dysfunction but also the means to re-establish mitochondrial calcium homeostasis.

## Supporting Information

S1 File Experimental Data. (XLS)
